# Bacterial interaction with host autophagy

**DOI:** 10.1080/21505594.2019.1602020

**Published:** 2019-04-12

**Authors:** Yao-Wen Wu, Fu Li

**Affiliations:** a Department of Chemistry, Umeå Centre for Microbial Research, Umeå University, Umeå, Sweden; b Chemical Genomics Centre of the Max Planck Society, Dortmund, Germany; c Max Planck Institute of Molecular Physiology, Dortmund, Germany

**Keywords:** Intracellular bacteria, autophagy, autophagosome, bacterial effectors, autophagy pathways

## Abstract

Autophagy is a conserved and fundamental cellular process mainly to recycle or eliminate dysfunctional cellular organelles or proteins. As a response to cellular stress, autophagy is used as a defense mechanism to combat the infection with pathogenic bacteria. However, many intracellular bacteria have developed diverse mechanisms to evade recognition, to manipulate the autophagic pathway, and to hijack the autophagosomal compartment for replication. In this review, we discuss recent understandings on how bacteria interact with host autophagy.

Macroautophagy (hereafter autophagy) is an evolutionarily conserved intracellular pathway that delivers bulk cytoplasmic components to lysosomes for degradation. The process involves the formation of double-membrane vesicle structures, termed autophagosomes, which sequester and engulf cytoplasmic components constitutively or upon stress. The subsequent autophagosome maturation is achieved by fusing with lysosomes, leading to the exposure of the cargo to lysosomal hydrolases for digestion. Cells employ this mechanism to maintain cellular homeostasis by constitutively removing protein aggregates, damaged organelles, and long-lived proteins. In response to cellular stresses, such as nutrient deprivation, oxidative stress, hypoxia, and pathogen invasion, autophagy can be rapidly induced for cellular adaptation to environmental changes. Autophagy plays an important role in cellular physiology including cell development and has been associated with diverse human diseases, including cancer, neurodegeneration and pathogen infection [1,2].

Autophagy can be non-selective or selective. Selective autophagy is involved in degradation of specific cargos, including aggrephagy, lipophagy, lysophagy, mitophagy, pexophagy and xenophagy. Recent advances suggest that autophagy plays a crucial role in the elimination of invading microorganisms (xenophagy), including intracellular bacteria that escape into the cytosol and those in damaged bacteria-containing vacuoles or phagosomes. On the other hand, bacteria have also evolved diverse mechanisms to avoid autophagy [3–6]. In this review, we focus on recent progresses on understanding interactions between bacteria (bacterial factors) and host autophagy proteins/pathways.

## Autophagy mechanisms

Autophagy is a complex process involving a few steps: initiation, nucleation, and formation of phagophore, phagophore elongation, fusion of autophagosome with lysosome and degradation in autolysosome. It is tightly regulated by a set of signaling pathways and involves a series of events, including changes in the level of autophagy-related (ATG) proteins, alterations in subcellular localization and post-translational modifications [7]. Yeast genetic studies have led to the identification of 36 ATG proteins that are hierarchically organized in function. The 15 core ATG proteins (ATG1-10, 12–14, 16, 18) involved in autophagosome biogenesis are highly conserved in mammals [8]. Two key upstream regulators are involved in the regulation of autophagy, including the energy sensors the mammalian target of rapamycin complex 1 (mTORC1) and AMP-activated kinase (AMPK). Under nutrient-rich conditions, mTORC1 is active and suppresses autophagy through phosphorylation of the unc-51-like kinase ULK (ATG1 in yeast). Under stress conditions such as nutrient deprivation (starvation), mTORC1 is inactive, allowing for activation of ULK by activated AMPK through the phosphorylation at another serine sites [9–11]. This leads to translocation of the ULK complex (ULK, FIP200, ATG101, and ATG13) to a certain domain of the endoplasmic reticulum (ER) membrane. The ULK complex regulates the class III phosphatidylinositol 3-kinase (PI3KC3) complex (Beclin1, ATG14L, VPS15, VPS34). Activation of the ULK complex is required for the phosphorylation and recruitment of the PI3KC3 complex, which leads to the production of phosphatidylinositol 3-phophate (PI3P) and initiation of phagophore (or isolation membrane) formation at ER-mitochondria contact sites [12–14]. Formation of PI3P recruits PI3P-binding proteins WD-repeat protein interacting with phosphoinoside (WIPI) to facilitate phagophore maturation. WIPI2 is involved in the recruitment of ATG16L1 in complex with ATG5-ATG12, which is required for the conjugation of ATG8 homologs, microtubule-associated protein light chain 3 (LC3) protein family, to phosphatidylethanolamine (PE) [15]. ATG9, the only transmembrane protein among the core ATG proteins, is implicated in tethering and fusion of small vesicles to the forming autophagsome [16–18]. Various intracellular membranes including the ER, the Golgi, mitochondria, the plasma membrane, and endosomes have been indicated as membrane sources for the expansion of nascent autophagosomes [19].

Two ubiquitin-like conjugation (UBL) systems control the production of the ATG12-ATG5-ATG16L1 complex and LC3-PE, which are crucial for the elongation and closure of phagophore [20,21]. Newly synthesized LC3 is processed by a protease, ATG4, to expose a C-terminal glycine. The resulting LC3 is conjugated to PE in a UBL reaction catalyzed by ATG7, ATG3 and the ATG12-ATG5-ATG16L1 complex. In another UBL system, ATG12 is conjugated to the lysine side chain of ATG5 in sequential reactions catalyzed by ATG7 and ATG10. The ATG12-ATG5 conjugate further forms a complex with Atg16L [22]. The ATG12-ATG5 conjugate promotes LC3-PE formation [23]. Atg8/LC3-PE can promote membrane tethering and hemifusion [24–26]. Atg4 releases lipidated LC3 proteins from the surface of closed autophagosomes, thus aid the recycling of LC3 for the generation of new autophagosomes [27,28]. Finally, with the involvement of an array of factors such as Rab7, syntaxin 17, VAMP8 and LAMP2, autophagosomes fuse with lysosomes to form degradative autolysosomes [29–31].

## Xenophagy

Studies over 15 years indicate that cells can also utilize autophagy to eliminate invading pathogens, which is referred to as xenophagy [32–34]. Xenophagy is one type of selective autophagy, which also requires the core autophagy machinery. Ubiquitination is often used as a mechanism for targeting cargos to selective autophagy including xenophagy. The cargo selection in selective autophagy also requires cargo receptors and adaptor proteins. These cargo-specific receptors usually contain two crucial domains, the ubiquitin-binding domain (UBD) and LC3-interacting region (LIR) motif, which are important for cargo recognition and interaction with the LC3 proteins, respectively. The receptor binds to ubiquitinated pathogens through its UBD and recruits them to the autophagosome membrane via the interaction of the LIR motif with LC3 proteins. For instance, upon infection of epithelial cells, *Salmonella Typhimurium* resides in the *Salmonella*-containing vacuole (SCV). However, damage of the SCV membrane by type III secretion system (T3SS) pore-forming activities on the membrane leads to a leakage of bacteria into the cytosol. Some of them are coated by ubiquitin and bind to autophagy receptors, which trigger the formation of LC3-positive nascent autophagosomes surrounding the bacteria [35]. Ubiquitination of bacteria can also be triggered by another post-translational modification, *S*-guanylation, in which 8-nitroguanosine 3ʹ,5ʹ-cyclic monophosphate (8-nitro-cGMP) modifies Cys residues of Group A *Streptococcus* (GAS) surface proteins. This process is regulated by nitric oxide (NO) signaling. *S*-guanylation promotes K63-linked ubiquitination at the cytosolic GAS surface and targets GAS to autophagosomes [36].

**Figure 1.**
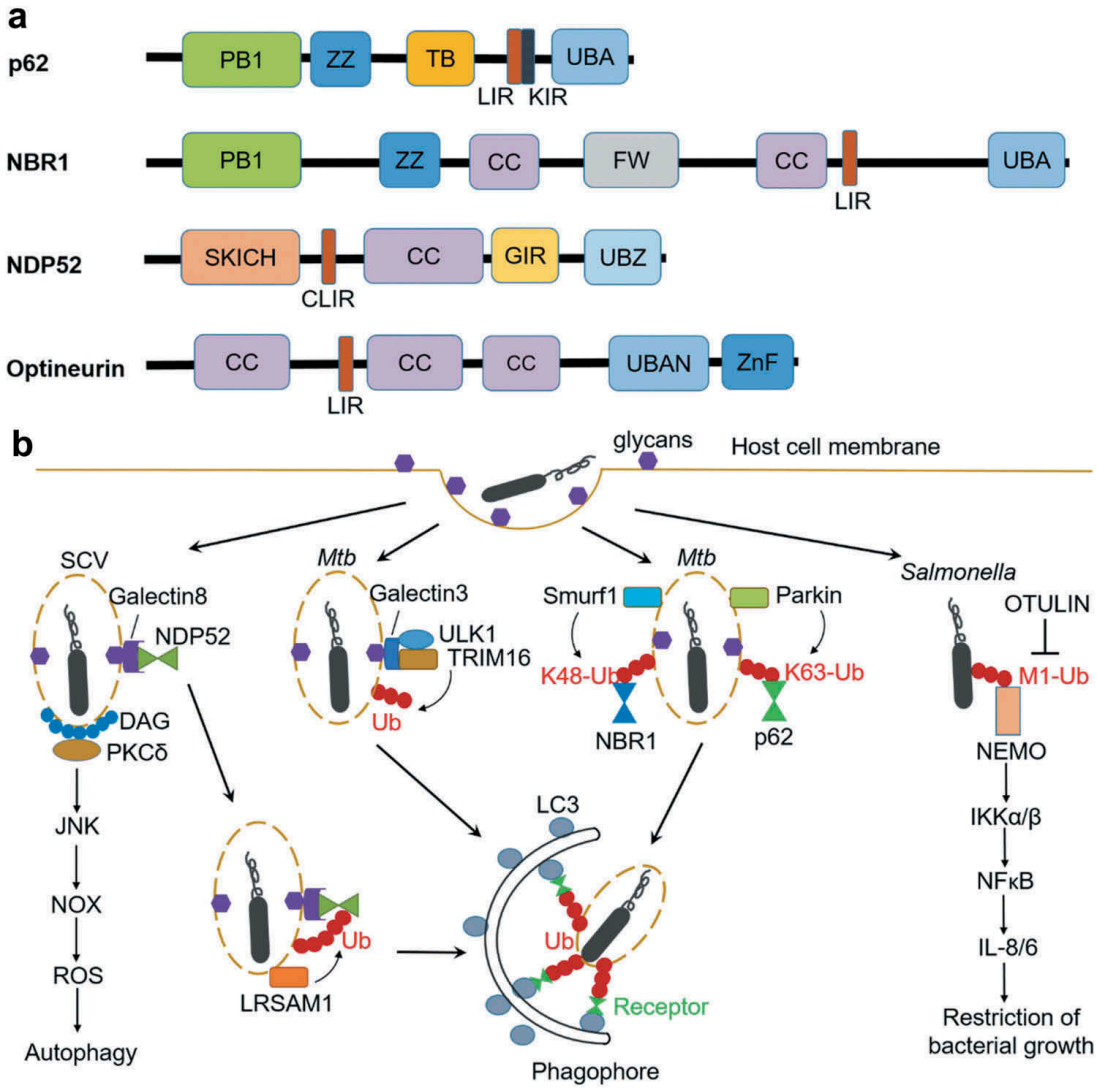
Anti-bacterial autophagy. (a) Domains of autophagy receptors. PB1, Phox and Bem1p domain; ZZ, ZZ-type zinc finger domain; TB, TRAF6-binding region; LIR, LC3-interacting region; KIR, Keap1-interacting region, UBA, ubiquitin-associated; FW, four W domain; SKICH, skeletal muscle and kidney-enriched inositol phosphatase carboxyl homology; CLIR, non-canonical LIR; CC, coiled-coil; GIR, galectin-8 interacting region; UBZ, ubiquitin-binding zinc finger; UBAN, ubiquitin-binding in ABIN and NEMO; ZnF, Zn-finger. (b) Ubiquitin-dependent and ubiquitin-independent pathways involved in autophagic elimination of intracellular *Mtb* and *Salmonella*. NOX, NADPH oxidase; ROS, reactive oxygen species; OTULIN, ovarian tumor (OTU) domain-containing deubiquitinase (also known as Gumby and Fam105b), IKK, IκB kinase, IL, interleukin.

As the first mammalian selective autophagy receptor identified, p62/SQSTM1 targets protein aggregates, peroxisomes, mitochondria, intracellular bacteria and other cargoes to autophagosomes [37–39]. Other known receptors include neighbor of BRCA1 gene 1 (NBR1), optineurin (OPTN) and nuclear domain 10 protein 52 (NDP52) (Figure 1(a)) [40–42]. p62, NDP52, and optineurin recognize ubiquitinated *S. Typhimurium* and target them to autophagosomes [39,41,42]. p62 and NDP52 are involved in the xenophagy of *Shigella flexneri* [43]. p62, NDP52 and NBR1 are required for delivery of *Mycobacterium tuberculosis* to autophagosomes [44,45].

Although the identity of the proteins targeted by ubiquitination remains elusive, several E3 ligases have been identified in recent studies (Figure 1(b)). The E3 ligase leucine-rich repeat and sterile α motif-containing-1 (LRSAM1) was shown to be important for ubiquitination upon *Salmonella* infection [46]. Ubiquilin1 (UBQLN1), a member of a protein family that contains an ubiquitin-like domain and an ubiquitin-associated domain, promotes IFN-γ-mediated autophagic clearance of *M. tuberculosis*. The function of Ubiquilin1 in xenophagy may be attributed to the recruitment or activation of an E3 ligase [47]. TRIM16, a tripartite motif (TRIM) protein containing E3 ligase domain, recognizes endomembrane damage through interactions with a glycan-binding lectin protein (Galectin3) in a ULK1-dependent manner (Figure 1(b)). TRIM16 controls ubiquitination of damaged endomembranes and associates with the core autophagy machinery ULK1, Beclin1, and ATG16L1. The cooperation between TRIM16 and Galectin3 in activation of autophagy protects cells from *M. tuberculosis* invasion [48]. The E3 ligase Parkin and Smurf1 are required for ubiquitination at K63 and K48, respectively, in xenophagy of *M. tuberculosis* (Figure 1(b)) [45,49]. In addition, Smurf1 also mediates K48-linked ubiquitination of *Listeria monocytogenes*, and restricts the proliferation of *Listeria* in macrophages [45]. Different types of polyubiquitin chains play distinct roles in bacterial autophagy by recruitment of different downstream factors. Indeed, p62 preferentially binds to K63 chains over K48 chains [50]. Smurf1-mediated K48-linked ubiquitination but not Parkin-mediated K63-linked ubiquitination is required for recruitment of proteasomes to bacteria-associated structures [45]. Liner polyubiquitin chain (M1-linked) decorated at the surface of cytosolic *S. Typhimurium* recruits NFκB essential modulator (NEMO), resulting in activation of IKKα/IKKβ and eventually NFκB, which promotes secretion of pro-inflammatory cytokines and restricts bacterial growth (Figure 1(b)) [51].

In addition to ubiquitin-dependent pathway, other ubiquitin-independent pathways also play a role in bacterial targeting during xenophagy, including nucleotide-binding oligomerization domain-containing (NOD) proteins, galectin, diacylglycerol (DAG) and the complement protein C3. The cytosolic NOD-like receptor (NLR) proteins NOD1 and NOD2 can detect peptidoglycans on intracellular bacteria such as *S. flexneri* and *L. monocytogenes*, and recruit ATG16L1 to the bacterial entry site at the plasma membrane [52,53]. Galectin8 accumulates on the damaged *Salmonella*-containing vacuoles via binding host glycans. NDP52 is recruited to SCVs initially through transient interaction with Galectin8 and subsequently by stable binding to ubiquitin (Figure 1(b)). Therefore, host cells employ Galectin8 as an early-stage danger receptor to detect vesicle-damaging pathogens [54]. Lipid second messenger DAG accumulates at the damaged SCV membrane when cells are invaded by *Salmonella*. DAG recruits PKCδ, which activates autophagy via the JUN N-terminal kinase (JNK) and NADPH oxidase pathways. Moreover, since bacteria-containing autophagosomes colocalize with either DAG or ubiquitinated proteins, both pathways could act independently to promote antibacterial autophagy (Figure 1(b)) [55]. Recent findings suggest that the interaction between the complement C3 protein and ATG16L1 plays an important role in xenophagy initiation. The C3-coated *L. monocytogenes* were targeted by autophagy through the C3-ATG16L1 interaction, leading to bacterial growth restriction. However, *S flexneri* and *S. Typhimurium* express outer membrane proteases omptins that cleave C3, thereby escape C3-mediated autophagy restriction [56].

## Bacteria suppress autophagy initiation

**Figure 2.**
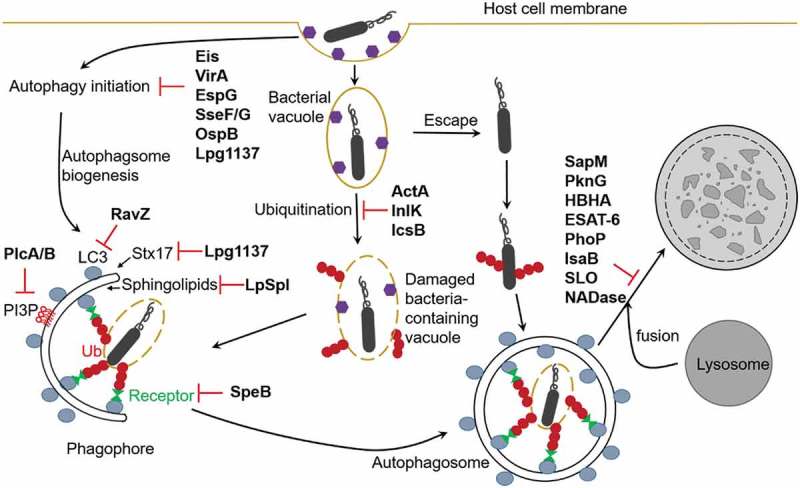
Manipulation of autophagy pathways by bacterial factors. Intracellular bacteria have evolved mechanisms to escape host autophagy in order to survive and replicate in host cells. Eis (*Mtb*), VirA and OspB (*Shigella*), EspG (EPEC), SseF/G (*Salmonella*), and Lpg1137 (*Legionella*) inhibit autophagy induction. RavZ, Lpg1137, and LpSpl (*Legionella*), and PlcA/B (*Listeria*) inhibit autophagosomes formation, SpeB (GAS), ActA and InlK (*Listeria*), and IcsB (*Shigella*) prevent recognition by host autophagy. SapM, PknG and HBHA (*Mtb*), ESAT-6 and PhoP (*Mtb* H37Rv), IsaB (*S. aureus*), SLO and NADase (GAS) inhibit autophagosome-lysosome fusion.

While some bacteria are targeted for lysosomal degradation by autophagy, other intracellular bacteria have evolved mechanisms to escape this defense system to survive and replicate in host cells (Figure 2, Table 1). Some bacteria escape host autophagy through the inhibition of autophagy induction. *S. Typhimurium* inhibits autophagy initiation *via* regulation of the AMPK-dependent activation pathway of mTOR. *S. Typhimurium* infection induces lysosomal degradation of Sirt1, LKB1 and AMPK in a *Salmonella* pathogenicity island 2 (SPI2) T3SS dependent manner. Therefore, SPI2 encoded virulence factors dismantle the AMPK activation complex, leading to activation of mTOR and subsequent inhibition of autophagy [57]. Effectors SseF and SseG secreted by *S. Typhimurium* inhibit autophagy initiation by direct interaction with host Rab1A GTPase. This interaction disrupts the interaction with its guanine nucleotide exchange factor (GEF), the TRAPPIII complex, and therefore Rab1A activation, leading to inhibition of the recruitment and activation of ULK1 [58]. Another example of bacterial effectors that target Rab1 is *S. flexneri* T3SS effector VirA and extracellular Enteropathogenic *Escherichia coli* (EPEC) effector EspG. VirA and EspG contain a GTPase activating protein (GAP) domain, which specifically inactivate Rab1 and leads to inhibition of autophagy induction [59].

**Table 1.** Mechanisms involved in the interaction of intracellular bacteria with host autophagy.

Bacterium	Effectors	Host targets	Function	Refs.
*Shigella flexneri*	IcsB	ATG5 Small GTPases SNARE CHMP5 (ESCRT-III complex)	Preventing ATG5 recognition of IcsA; counteracting recruitment of Tecpr1, spetins, ubiquitin, p62 and NDP52; interfering with membrane trafficking	[43,79–82]
	IcsB	Toca-1	Preventing recognition by NDP52	[95]
	VirA	Rab1A	Inactivating Rab1 as a GAP, inhibiting autophagy induction	[59]
	OspB	IQGAP1	Activating mTORC1	[96]
*Legionella pneumophila*	RavZ	Ubiquitin?	Preventing ubiquitin recruitment	[72]
	RavZ	LC3-PE	Deconjugating LC3-PE, inhibiting autophagosome formation	[67,69,70]
	LpSpl	Sphingolipids	Interfering with host sphingosine biosynthesis	[74]
	SidE	Rag GTPases	Inhibiting mTORC1 via ubiquitination of Rag	[97]
	Lgt	eEF1A, mTORC1	Inhibiting protein synthesis and activating mTORC1 via liberation of amino acids	[97]
	Lpg1137	Stx17	Depleting Stx17, inhibiting autophagosome biogenesis	[73]
*Mycobacterium tuberculosis*	Eis	MKP-7, JNK	Acetylating and activating MKP-7 that inactivates JNK, inhibiting autophagy induction	[61]
	SapM, PknG	Rab5 and Rab7	Inhibiting Rab5-Rab7 exchange and autophagosome maturation	[75]
	CpsA	NADPH oxidase	Inhibiting recruitment of *Mtb* to LC3-associated phagosomes	[98]
	HBHA	LC3	Inhibiting LC3 expression and the maturation of autophagosomes	[99]
*M. tuberculosis* H37Rv	PhoP and ESAT-6	Rab7	Inhibiting autophagosome maturation	[76]
*Mycobacterium marinum*	ESX-1	TORC1	Blocking the autophagic flux	[100]
*Listeria mono-cytogenes*	ActA	Arp2/3	Catalysing actin polymerization on the bacterial surface to disguise from autophagic recognition	[83,84]
	InlK	MVP	Preventing cytosolic bacteria from autophagic recognition	[85]
	PlcA, PlcB	PI3P	Reducing PI3P levels to inhibit pre-autophagosomal structures	[65]
*Salmonella Typhimurium*	SseF and SseG	Rab1A-TRAPPIII	Disrupting Rab1A-TRAPPIII interaction and therefore Rab1A activation, inhibiting autophagy induction	[58]
	SPI2	Sirt1, LKB1, AMPK	Inducing lysosomal degradation of Sirt1, LKB1 and AMPK, activating mTOR, inhibiting autophagy initiation	[57]
*Staphylococcus aureus*	Hla	Unknown	Activating autophagy pathway independent of PI3KC3 and Beclin1	[88]
	IsaB	Unknown	Inhibiting autophagsome maturation	[89]
	Unknown	MAPK14, ATG5	Inibiting autophgosome maturation	[90]
Group A *Streptococcus (GAS)*	SpeB	p62, NDP52, NBR1	Degrading autophagy receptors to prevent recognition by host autophagy	[86]
	Streptolysin O (SLO), NADase	Unkown	Preventing maturation of the GAS-containing autophagosome-like vacuoles	[101]
*Francisella tularensis*	Type VI secretion system	Beclin1	Hijacking autophagy machinery to derive nutrients for replication in an ATG5-independent autophagy pathway	[102]
*Escherichia coli O157:H7*	Tir	PKA	Activating protein kinase A	[103]
*Burkholderia pseudomallei*	BopA	Unknown	23% identity with IcsB of *Shigella*	[104]

*M. tuberculosis* str. H37Rv via its effector protein enhanced intracellular survival (Eis) inhibits autophagy induction by disruption of JNK-ROS (reactive oxygen species) signaling pathway [60]. Eis was found to be an N-acetyltransferase that mediates acetylation and activation of a JNK-specific phosphatase mitogen-activated protein kinase phosphatase-7 (MKP-7), which inactivates JNK [61].

Some extracellular bacterial toxins inhibit autophagy induction through increasing the level of cyclic AMP (cAMP), a second messenger with roles in many cellular processes, which negatively regulates autophagy. Two cAMP-elevating toxins, edema toxin (Edtx) from *Bacillus anthracis* and cholera toxin (Ctx) from *Vibrio cholerae*, have been shown to inhibit autophagy including antibacterial autophagy *via* the protein kinase A (PKA) pathway [62]. *Vibirio cholerae* MARTX toxin displays PI3P-seicific phospholipase A1 (PLA1) activity that reduces intracellular PI3P levels, leading to inhibition of autophagy and endosomal trafficking [63]. In contrast, *Helicobacter pylori* (*Hp*) vacuolating cytotoxin (VacA) induces host autophagy *via* inhibition of mTORC1 during *Hp* infection. VacA targets to mitochondria and impairs mitochondrial function, leading to disruption of cellular amino acid homeostasis and thereby inhibition of mTORC1 [64].

## Bacteria inhibit autophagosome formation

The core autophagy machinery is required for the elimination of intracellular bacteria. Some bacteria manipulate autophagy by directly interfering with the core autophagy machinery. Upon *L. monocytogenes* infection of epithelial cells, *Listeria* effector LLO, a pore-forming toxin, triggers a rapid induction of host amino acid starvation, characterized by the inhibition of mTOR and the upregulation of the starvation kinase GCN2-dependent integrated stress response (ISR) pathway [65]. Very recent work showed that LLO induces non-canonical autophagy involving recruitment of LC3 on the damaged *Listeria*-containing vacuole in a ULK-independent manner. But this process has not apparent role in restricting bacterial growth [66]. However, two *Listeria* phospholipases PlcA and PlcB reduce PI3P levels, leading to inhibition of pre-autophagosomal structures and preventing efficient targeting of cytosolic bacteria by xenophagy [65].

*Legionella pneumophila* inhibits autophagy by injecting a T4SS effector protein called RavZ into the host cytoplasm. RavZ functions as a cysteine protease and irreversibly deconjugates LC3 proteins from PE to inhibit autophagosome formation [67]. Unlike Atg4 that cleaves the amide bond between terminal glycine and PE, RavZ cleaves the amide bond before glycine. As a consequence, the RavZ-cleaved LC3 proteins cannot be relipidated, leading to inhibition of autophagosome formation. A study combining chemical and structural approaches demonstrated that RavZ extracts LC3-PE from the membrane before deconjugation [68,69]. RavZ targets autophagosomal membrane via the C-terminal PI3P-binding domain and the α3 helix and recognizes the LC3 molecule on the membrane via its N-terminal LIR motif [69–71]. The RavZ α3 helix is involved in the extraction of the PE moiety and docking of the acyl chains into the lipid-binding site of RavZ that is related in structure to that of the phospholipid transfer protein Sec14 [69]. Recent study showed that RavZ interferes with ubiquitin recruitment to the *Salmonella*-containing vacuoles. The inhibitory effect is dependent on the proteolytic activity of RavZ [72].

Another *Legionella* effector Lpg1137 is a serine protease that degrades syntaxin 17 (Stx17), a soluble N-ethylmaleimide-sensitive factor attachment protein receptor (SNARE) protein that has been shown to recruit the PI3KC3 complex to the mitochondria-associated ER membrane through association with ATG14L and to be involved in autophagosome-lysosome fusion [13,30]. Lpg1137 leads to depletion of Stx17 and reduction of PI3P formation, suggesting that *Legionella* can interfere with early stage of autophagosome biogenesis [73]. The *Legionella* effector sphingosine-1 phosphate lyase (LpSpl) inhibits autophagy through interfering with host sphingosine biosynthesis, which is crucial for autophagy. Structural analysis showed that SPLs are highly conserved from bacteria to human. LpSpl possesses S1P lyase activity that targets several sphingolipids critical for macrophage function and autophagy [74].

## Bacteria block autophagosome-lysosome fusion

Two *M.tuberculosis (Mtb*) effectors the phosphatase SapM and the kinase PknG that are exported by the SecA2-dependent protein export system play a role in inhibition of phagosome and autophagosome maturation, facilitating the survival and replication of *Mtb* in host cells. The phosphatase activity of SapM is required for inhibiting Rab5-Rab7 exchange, which is crucial for phagosome maturation [75]. Another *Mtb* H37Rv virulence factors PhoP and Esat-6 also play a role in blocking autophagosome maturation in macrophages and in dendritic cells by inhibiting Rab7 recruitment on Mtb-containing autophagosomes [76,77].

Bacterial inhibition of lysosomal acidification could induce bacterial expulsion, leading to relieving of bacterial load in infected cells. Upon infection of bladder epithelial cells (BECs), uropathogenic *E. coli* (UPEC) are targeted by autophagosomes and subsequently shuttled to multivesicular bodies (MVBs) to form amphisomes before ending up in lysosomes. But they are not degraded in lysosomes, as they can neutralize lysosomal pH. Mucolipin TRP channel 3 (TRPML3), a transient receptor potential cation channel at the lysosome, triggers Ca^2+^ efflux and initiates lysosome exocytosis, leading to the expulsion of exosome-encased bacteria. Interestingly, the bacterial expulsion requires autophagy machinery. Autophagy induction by an autophagy-inducing peptide reduces the bacterial burden of infected cells [78]. This study suggests that anti-bacterial autophagy does not necessary end up with autophagic degradation and could also help to expel bacteria from host cells.

## Bacteria escape from recognition by autophagy

Some bacteria mask themselves to avoid recognition by autophagy machinery. The *S. flexneri* surface protein IcsA (also known as VirG) can be recognized by ATG5 to trigger xenophagy, which requires a host protein tectonin domain-containing protein (Tecpr1) that connects Atg5-targeted bacteria with the WIPI2-positive phagophore membrane [79]. To battle the host defense system, *S*. flexneri secretes IcsB that competes with ATG5 for binding to IcsA, thus escapes from recognition by autophagy [80]. Compared to wild type bacteria, Δ*icsB* mutants were more ready to be entrapped by cage-like structures formed by septins, which facilitate recruitment of ubiquitin, p62, and NDP52 to target the bacteria for autophagy [43,81]. A very recent study showed that IcsB is a C18 fatty acyltransferase that mediates lysine N^ϵ^-fatty acylation. About 60 targets are modified by IcsB during infection, including small GTPases (Ras, Rho and Rab family proteins) and SNARE proteins. One of the targets CHMP5, a component of the endosomal ESCRT-III complex, is required for anti-*Shigella* autophagy, suggesting that IcsB acts on the trafficking of endosome-derived *Shigella*-containing vacuoles and IcsB-mediated inhibition of autophagy is an indirect result of CHMP5 inactivation [82].

During *L. monocytogenes* infection, ActA at the bacterial surface recruits the actin-related protein (Arp2/3) complex, which catalyzes actin polymerization on the bacterial surface and disguises the bacteria from autophagic recognition [83,84]. *L. monocytogenes* virulence factor InlK interacts with host major vault protein (MVP), the main component of cytoplasmic ribonucleoprotein particles named vaults, which prevents cytosolic bacteria from autophagic recognition. InlK rapidly recruits MVP at the surface of the bacterium. Subsequently, ActA replaces InlK, leading to a switch of the shield from MVP to actin [85].

Some bacteria escape from autophagy by targeting autophagy receptors instead of forming a shield. One example is Group A *Streptococcus* (GAS). M1T1 clone of GAS expresses cysteine protease SpeB that degrades p62, NDP52, and NBR1 in the host cell, preventing recognition by host autophagy [86].

## Bacteria hijack autophagy for replication

Some bacteria use the autophagy machinery to form their replicative niche, which is like an autophagosome but does not fuse with the lysosome. One example is *Staphylococcus aureus*, which is sequestered in double-membrane autophagosomes during infection. *S. aureus* is not able to replicate in autophagy-deficient cells. After replication, *S. aureus* escapes from autophagosomes into the cytoplasm, which results in ATG5-dependent host cell death [87]. The *S. aureus* pore-forming toxin α-hemolysin (Hla) was shown to be required for the activation of the autophagy pathway independent of PI3KC3 and Beclin1 [88]. *S. aureus* inhibits autophagosome-lysosome fusion through the expression of immunodominant surface antigen B (IsaB) protein [89]. However, a recent study argued for the role of autophagy in *S. aureus* replication. It was shown that replication of *S. aureus* inside nonprofessional phagocytes is independent of autophagy. Intracellular *S. aureus* is ubiquitinated and recognized by autophagy receptors p62, NDP52 and optineurin, leading to phagophore recruitment. But *S. aureus* is able to evade autophagic degradation through activation of MAPK14, ATG5 phosphorylation, and inhibition of fusion with lysosomes [90].

Other examples include *Anaplasma phagocytophilum* [91], *Coxiella burnetii, Brucella spp., Francisella tularensis*, and *Yersinia pseudotuberculosis* [92], which manipulate the autophagy in host cells to acquire nutrient for their replication [93].

## Conclusion remarks

In the past 15 years, numerous studies have revealed the crucial function of autophagy (xenophagy) in innate immunity targeting intracellular bacterial pathogens. The interaction between bacterial pathogens and host autophagy is a mutual process. On one hand, bacteria can be restricted and eventually eliminated by autophagy. On the other hand, pathogenic bacteria have evolved many mechanisms to escape, subvert or even hijack the host autophagy machinery. Fundamental questions in these areas concern how intracellular bacteria are targeted by xenophagy and how bacteria manipulate host autophagy.

To address the former question, future research would elucidate the signaling pathways involved in xenophagy induction, the nature of ubiquitinated proteins associated with invading bacteria, the Ub ligases involved in ubiquitination, other non-Ub factors or Ub-receptors-LC3 independent pathways involved in bacterial recognition, etc. To address the latter question, future endeavor would identify novel bacterial effectors and their host targets, and the mechanisms how they interact with each other. Moreover, non-canonical autophagy pathways that are independent of some of the core autophagy machinery and do not require formation of double-membrane autophagosomes have emerged as an important anti-bacterial mechanism, such as LC3-associated phagocytosis (LAP) [94]. It is interesting to see if there are more extracellular bacterial toxins that interfere with host autophagy [62,64]. A better understanding of the mechanisms underlying the interaction of bacterial with host autophagy will eventually benefit the development of a new therapeutic intervention for bacterial infections.
